# Supplementary material for: Improving dog training methods: Efficacy and efficiency of reward and mixed training methods
Source: PLoS One. 2021 Feb 19;16(2):e0247321. doi: 10.1371/journal.pone.0247321 (PMC7895348; doi:10.1371/journal.pone.0247321)
Supplement: S1 Annex — (DOCX) [file pone.0247321.s001.docx]

S1 Annex. Qualitative scoring system for the test for efficacy evaluation.

**Exercise 1. Food refusal**

Willingness

- Outstanding (Dog has a positive attitude and shows the correct level of motivation for the exercise)
- Sufficient (Dog has a neutral attitude but does not compromise the exercise)
- Insufficient

Position

- Outstanding (Dog remains in the designated place and body position when instructed)
- Sufficient (Dog moves slightly [not more than a body length] from the designated place or changes body position)
- Insufficient

Response

- Outstanding (Dog remains indifferent towards the food)
- Sufficient (Dog hesitates, may eventually smell or touch in the pieces of food, but does not eat any)
- Insufficient

**Exercise 2. Interrupted recall**

Willingness

- Outstanding (Dog has a positive attitude and shows the correct level of motivation for the exercise)
- Sufficient (Dog has a neutral attitude but does not compromise the exercise)
- Insufficient

Response

- Outstanding (Dog stops immediately when instructed)
- Sufficient (The stop response is not immediate but dog stops before reaching the handler)
- Insufficient

**Exercise 3. Dumbbell retrieval**

Willingness

- Outstanding (Dog has a positive attitude and shows the correct level of motivation for the exercise)
- Sufficient (Dog has a neutral attitude but does not compromise the exercise)
- Insufficient

Response

- Outstanding (Dog performs the desired behaviors [sit, go to dumbbell, pick, retrieve, sit and release] on handler’s cues)
- Sufficient (Dog performs the desired behaviors with some mistakes but does not compromise the dumbbell retrieve)
- Insufficient

**Exercise 4. Placing items in basket**

Willingness

- Outstanding (Dog has a positive attitude and shows the correct level of motivation for the exercise)
- Sufficient (Dog has a neutral attitude but does not compromise the exercise)
- Insufficient

Response

- Outstanding (Dog places all items in the basket immediately after cued)
- Sufficient (Dog makes some mistakes but successfully places all the items in the basket)
- Insufficient

**Exercise 5. Surprise exercise**

Willingness

- Outstanding (Dog has a positive attitude and shows the correct level of motivation for the exercise)
- Sufficient (Dog has a neutral attitude but does not compromise the exercise)
- Insufficient

Position (To be evaluated only for the food refusal component of the exercise)

- Outstanding (Dog remains seated by the handler’s side)
- Sufficient (Dog moves slightly [not more than a body length] from the designated place or changes body position)
- Insufficient

Response

- Outstanding (Dog remains indifferent towards the food + Dog performs the desired behaviors [sit, go to dumbbell, pick, retrieve, sit and release] on handler’s cues)
- Sufficient (Dog hesitates, may eventually smell or touch in the pieces of food, but does not eat any + Dog performs the desired behaviors [sit, go to dumbbell, pick, retrieve, sit and release] with some mistakes but does not compromise the dumbbell retrieve)
- Insufficient

Further instructions:

If the handler has to repeat a cue once, the exercise can have a maximum score of “sufficient”. If a third cue is required, the exercise is immediately scored as “insufficient”.

If one of the aspects evaluated (willingness, position, response) receives “insufficient”, the final score for the exercise is “insufficient”. To decide between a final score of “sufficient” or “outstanding” the following rules apply:

For the exercises with an odd number of aspects being evaluated (Exercises 1 and 5), the final score will be determined by the score that was attributed more often. For example, if, in Exercise 1, the dog receives “sufficient” for willingness and “outstanding” for position and response, the final score should be “outstanding”. For the exercises with an even number of aspects under evaluation (Exercises 2, 3 and 4), if there is a draw, the aspect ‘response’ should be given more weight. For example, if, in Exercise 2, the dog receives “sufficient” for willingness and “outstanding” for response, the final score should be “outstanding”.
