# Supplementary material for: Improving dog training methods: Efficacy and efficiency of reward and mixed training methods
Source: PLoS One. 2021 Feb 19;16(2):e0247321. doi: 10.1371/journal.pone.0247321 (PMC7895348; doi:10.1371/journal.pone.0247321)
Supplement: S2 Annex — (DOCX) [file pone.0247321.s002.docx]

S2 Annex. Quantitative scoring system for the test for efficacy evaluation.

**Exercise 1. Food refusal (0-10)**

Dog remains indifferent towards the two pieces of food – 10

Dog hesitates, smells, touches or licks one of the two pieces of food, but does not eat – 7.5

Dog hesitates, smells, touches or licks the two pieces of food, but does not eat any – 5

Dog eats one piece of food – 2.5

Dog eats both pieces of food - 0

**Exercise 2. Interrupted recall (0-10)**

Dog recalls in a fast trot or running after first recall cue and stops immediately after the first cue to stop – 10

Dog recalls in a fast trot or running within two recall cues, but shows a slight delay to stop after the first stop cue – 7.5

Dog recalls in a fast trot or running within two recall cues and stops immediately or shows only a slight delay to stop after a second stop cue – 5

Dog recalls in a slow pace or; Dog recalls in a fast trot or running but shows a long delay to stop within two stop cues – 2.5

Dog does not respond to recall cue within two attempts, anticipates the stop cue or does not stop within two stop cues - 0

**Exercise 3. Dumbbell retrieval (0-10)**

On first retrieve cue, dog moves towards the dumbbell, picks it up and brings it to the handler, sitting in front of him/her, only releasing on cue – 10

Dog drops the dumbbell while retrieving but picks it up on its own initiative and successfully finishes the exercise or; Dog sits in front of handler but releases before release cue is given – 7.5

Two retrieve cues are needed for the dog to first pick up the dumbbell but then dog successfully finishes the exercise or; Dog drops the dumbbell besides the handler and the handler can pick it up without taking a step - 5

Dog drops the dumbbell besides the handler but the handler cannot pick it up without taking one step – 2.5

Dog does not pick the dumbbell within two retrieve cues, or drops it and does not pick it up again - 0

**Exercise 4. Placing items in basket (0-10)**

On first cue, dog places all items within the basket; dog does not chew or play with the items – 10

Dog drops at least one item but picks it up on its own initiative and successfully finishes the exercise or; Dog chews or plays with the items – 7.5

A second cue is needed for the dog to complete the exercise - 5

Some items are placed outside the basket but within a 50 cm radius around the basket – 2.5

Dog fails to pick all items within two cues or the items are placed more than 50 cm away from the basket – 0

**Exercise 5. Surprise exercise (0-10)**

Dog remains indifferent towards the two pieces of food AND On first retrieve cue, dog moves towards the dumbbell, picks it up and brings it to the handler, sitting in front of him/her, only releasing on cue – 10

Dog hesitates, smells, touches or licks one of the two pieces of food, but does not eat AND/OR Dog drops the dumbbell while retrieving but picks it up on its own initiative and successfully finishes the exercise or; Dog sits in front of handler but releases before release cue is given – 7.5

Dog hesitates, smells, touches or licks the two pieces of food, but does not eat any AND/OR Two retrieve cues are needed for the dog to first pick up the dumbbell but then dog successfully finishes the exercise or; Dog drops the dumbbell besides the handler and the handler can pick it up without taking a step - 5

Dog eats one piece of food AND/OR Dog drops the dumbbell besides the handler but the handler cannot pick it up without taking one step – 2.5

Dog eats both pieces of food AND/OR Dog does not pick the dumbbell within two retrieve cues, or drops it and does not pick it up again - 0
