# Supplementary material for: Improving dog training methods: Efficacy and efficiency of reward and mixed training methods
Source: PLoS One. 2021 Feb 19;16(2):e0247321. doi: 10.1371/journal.pone.0247321 (PMC7895348; doi:10.1371/journal.pone.0247321)
Supplement: Attachment — Submitted filename: Response to reviewers.docx [file pone.0247321.s003.docx]

RESPONSE TO REVIEWERS

We appreciate all the constructive criticism provided by the two anonymous reviewers. In our opinion, the manuscript as it currently stands has improved substantially. In what follows, we present detailed responses to all the comments.

Editor

1. Please ensure that your manuscript meets PLOS ONE's style requirements, including those for file naming. The PLOS ONE style templates can be found at

<https://journals.plos.org/plosone/s/file?id=wjVg/PLOSOne_formatting_sample_main_body.pdf> and

<https://journals.plos.org/plosone/s/file?id=ba62/PLOSOne_formatting_sample_title_authors_affiliations.pdf>

Some changes were made and we believe the new version of the manuscript fully complies with PLOS ONE’s requirements.

2. Thank you for stating the following financial disclosure:

"The author(s) have applied to funding for this work and are currently waiting for decision."

At this time, please address the following queries:

a. Please clarify the sources of funding (financial or material support) for your study. List the grants or organizations that supported your study, including funding received from your institution.
b. State what role the funders took in the study. If the funders had no role in your study, please state: “The funders had no role in study design, data collection and analysis, decision to publish, or preparation of the manuscript.”
c. If any authors received a salary from any of your funders, please state which authors and which funders.
d. If you did not receive any funding for this study, please state: “The authors received no specific funding for this work.”

Please include your amended statements within your cover letter; we will change the online submission form on your behalf.

Please refer to Cover Letter. We have currently no funding approved for the study and hence the statement for financial disclosure at this time should be “The authors received no specific funding for this work.”

3. We note that you have stated that you will provide repository information for your data at acceptance. Should your manuscript be accepted for publication, we will hold it until you provide the relevant accession numbers or DOIs necessary to access your data. If you wish to make changes to your Data Availability statement, please describe these changes in your cover letter and we will update your Data Availability statement to reflect the information you provide.

We plan on submitting data as supporting information.

4. Please include captions for your Supporting Information files at the end of your manuscript, and update any in-text citations to match accordingly. Please see our Supporting Information guidelines for more information: <http://journals.plos.org/plosone/s/supporting-information>.

Done. Thank you for pointing that out!

**Reviewer #1:**

*1. Ethics statement: ethical approval of the study protocol is desirable.*

We have now submitted our protocol to both the Committee for Ethics and Responsible Conduct in Research (human subjects research) and from the Animal Welfare and Ethics Body (animal research) of i3S, University of Porto.

*2. Data availability: where will the data of the study be made available?* [*https://journals.plos.org/plosone/s/data-availability*](https://journals.plos.org/plosone/s/data-availability) *(there are some recommendations on this page)*

We plan on submitting data as supporting information.

*3. Abstract: you might prefer "randomized" instead of "pseudorandomized" to avoid confusion by readers without a scientific background?*

We have now realized that in the first version of the manuscript we did not explain what we meant by pseudo-randomization and, of course, that could be confusing for the readers (especially for those with no scientific background). In the current version of the manuscript, in the section “Subjects”, the following information can be found:

“A stratified randomization method [23] will be used to assign animals to the two groups. This method allows for balancing in terms of subjects’ baseline characteristics (covariates) that may potentially affect the dependent variables under study. In the present study the following covariates will be taken into account: dog sex, age, breed and previous training experience (obedience, odor detection, protection work).”

In the meantime, we will stick to the use of the word “pseudo-randomly” in the Abstract. In our opinion, using “randomly” can be deceptive, leading the readers to think that we will perform a true random allocation of dogs to the two groups.

*4. Line 57: what do you mean by "other non-invasive techniques (reward methods)"? It sounds a bit redundant as compared to line 56 ('exclusively rewards') but I may have missed something.*

With "other non-invasive techniques we meant extinction and negative punishment, other operant conditioning techniques (besides positive reinforcement) that are used within the scope of reward-based methods in dog training. However, we recognize that the way it was phrased could be confusing and we have removed the word “exclusively”. Now it reads:

“The methods used to train dogs range broadly with some using rewards and other non‑invasive techniques (reward methods), others using mainly aversive stimuli (aversive methods) and still other using a combination of both (mixed methods).”

*5. Line 63: in light of the potential importance of this large study, you could write its sample size, e.g. "of companion dog training (n=XXX)"*

We followed the reviewer’s suggestion and changed the text to:

“Recently, in the first large-scale quasi-experimental study of companion dog training (n=92), Vieira de Castro et al (accepted for publication) [2] found that dogs trained with aversive stimuli displayed more stress behaviors during training…”

*6. Line 75: just remove a space here "aversive/mixed methods"*

Done. Thank you!

*7. Line 114: in which context are they going to be video recorded? During the whole training process? Analysis of behaviour of both dog and handler?*

We now acknowledge that, in the first version of the protocol, this information was not made clear. The information can now be found in the following places throughout the protocol:

Line 113 Dogs and handlers will be video recorded for further analysis of behavior. Individual handlers will be identifiable from the video footage. Material in which individuals can be identified will only be used by the research team for research purposes (i.e., to control for the training techniques and for data analysis).

Line 197 all training sessions will be video recorded.

Line 247 The tests will be recorded

Line 258 two researchers blind to the experimental groups and to the goals of the study will analyze the videos of the tests using a quantitative scoring system

Line 263 The video recordings of the training sessions and the tests will also be used to assess dog welfare through the analysis of stress behaviors as in Vieira de Castro et al (accepted for publication). These will also allow for the analysis of handler behavior and other aspects of training such as the frequency, type and timing of the stimuli applied.

*8. Line 116: will this material remain confidential or be shared (e.g. supplementary material) upon study publication? To clarify, you could write "will only be used by the research team for research purposes (i.e. data analysis, XXX)".*

We will share untreated quantitative data but not the videos. In order to make this information clear, the text now reads: “Material in which individuals can be identified will only be used by the research team for research purposes (i.e., to control for the training techniques and for data analysis”.

*8. Lines 123-125: same comment from above about ethical approval*

See comment above.

*9. Lines 128-132; 204-210: besides randomization of individuals and control of the variables 'years of training', age and gender, are you going to use any practical measure to reduce group bias? If dogs are likely to differ in performance (e.g. due to age difference, genetics, training experience), a test of baseline performance (e.g. time required to learn a simple new task) could be added.*

Because, to our knowledge, there is no validated test for evaluating baseline performance, we will not use a test of this sort in order to allocate dogs to groups. However, as can now read in line 150 “as part of their certification process as working dogs, all the animals had to perform and pass the obedience component of a BH test [24]”. This, is our view, already ensures that all the animals have some equivalence in their baseline performance.

*10. Lines 159-160: are all dogs naive to these tasks?*

This is crucial information and it was missing in the previous version of the manuscript. We have added the following paragraph: “Despite all dogs being naïve to the specific exercises included in the present study (food refusal, interrupted recall, dumbbell retrieval and placing items in basket – the detailed description of the exercises is presented below), two similar behaviors are trained as part of the training programs of PSP and RPara. Namely, dogs are trained to retrieve a motivator (e.g., a tug or bite pad), although not to the formality and precision that is going to be required in the ‘dumbbell retrieve’ exercise, and they are also usually trained to interrupt a send away (i.e., they are trained to run forward to a motivator and interrupt the running when instructed). The ‘food refusal’ and ‘place items in the basket’ exercises are not part of the training programs and are thus new or near to completely new for all the animals. Because previous training on similar behaviors may have carryover effects on the training planned for the study, at the time of the beginning of the study, each participating dog’s training history will be thoroughly evaluated and, if needed, this will also be included as a covariate in the randomization process.”

*11. Lines 161-162: who will control the number of sessions and duration of sessions? Self-report, diary, video records? How long is each session expected to last? Are you going to standardize the duration of the sessions?*

We have added the following information to the text:

Line 190: “Training sessions will be conducted two days per week, with a gap between training days no longer than three days. Each training session will have a maximum duration of 10 minutes and up to six training sessions can be conducted per day. Within each training day, a break of at least 30 minutes between training sessions will be required.”

Line 196: “Information regarding the number of training sessions, their duration and the behaviors being trained will be annotated by each handler in a notebook (specifically designed for the study) for each training day. In addition, all training sessions will be video recorded.”

*12. Lines 165-166: at this task, wouldn't be important to tell the handlers to cover "all" the zones of this field as opposed to just letting them walk "randomly"?*

*Is the food going to be spilt on the same areas for all dogs?*

We decided to change this exercise to the food refusal exercise of the dog sport of Mondioring. This way, we have both a more standardized exercise and a stronger basis for the scoring.

*13. Lines 167-168: are they going to use only verbal commands or visual cues are also allowed? Standardization is important.*

Only verbal cues will be allowed. This is now clearly stated in line 234: “Only verbal cues will be allowed during the test.”

*14. Lines 169-174: same as above regarding visual and verbal cues.*

See response to previous comment.

*15. Line 174: is the food going to be placed on the same area for all the dogs?*

See response to comment 12 above.

*16. Any target to help the handlers throw the dumbbell on a similar location?*

Now line 219 reads: “With the dog sitting at his/her side, the handler throws the dumbbell to a distance of roughly 10 meters (marked in the floor in order to help) and then instructs the dog to retrieve it”.

*18. Line 183: the techniques allowed for each group should be included in the protocol. It lacks details on how exactly the group reward differs from the group mixed - this is a very important piece of information.*

We added a section entitled “Training methods” where this information is now presented. Thank you for pointing this out, this is definitely crucial information.

*19. Lines 188-190: who is going to score the dogs? Ideally, more than one person should rate the performances and inter-rater reliability should be calculated (especially for the 'general impression' score, as it is more subjective).*

This information can now be found in section “Data analysis”:

“Two different approaches will be used to analyze the performance of the dogs in the test. Three international experts on working dog training will be invited to assess dog performance *in situ* on the test days. The experts, who will be blind to the experimental groups and to the goals of the study, will be instructed to use a qualitative scoring system, according to which the dog performance for each exercise should be classified as ‘insufficient’, ‘sufficient’ or ‘outstanding’ (see Annex 1 for full details). Moreover, two researchers blind to the experimental groups and to the goals of the study will analyze the videos of the tests using a quantitative scoring system, following which the dog performance for each exercise will receive a score ranging from 0 to 10 (see Annex 2 for full details). Inter-observer reliability will be calculated for each exercise. The quantitative scoring system was developed based on FCI rules and guidelines for Obedience, Mondioring and IGP trials [24, 30, 31].”

***Reviewer #2:***

*This protocol covers an interesting and important applied topic: the efficiency and effectiveness of different dog training approaches. However, many methodological details are missing, making it impossible to assess the soundness of the proposed methods.*

*1. There are several different reward-based training methods and aversive training methods and, among a given category, they differ in their effectiveness. For example, research has shown that, among reward training methods, diverse methods differ in their efficiency (Fugazza and Miklósi 2014) and effectiveness (Fugazza and Miklósi 2015). I am not aware of studies comparing different aversive methods, but it is logical to assume that, for example, diverse aversive stimuli may differ in the intensity of their effects, at least, and potentially in other aspects. It is therefore crucial that the authors carefully describe the methods that will be used for training, rather than only classifying them as reward-based or aversive. A detailed description of the protocol applied in the training sessions with the two methods would help enormously in this sense.*

Please refer to response to comment #18 of Reviewer #1. We acknowledge that information on training methods was actually missing in the previous version of the manuscript and we have now added a section entitled “Training methods”, where we detail which procedures and tools can be used for each group. We will not use standardized protocols for training and the reasons for this choice are now underpinned in lines 175-180: “Some flexibility for choosing training equipment and procedures will thus be allowed (as opposed to have the handlers following previously defined and detailed training protocols). The reason for this decision is that this study aims to reflect a real-life situation of dog training, where different handlers use different approaches (within the same training method – reward or mixed) and, especially, where the individual dog and its natural tendencies and behaviors usually dictate the training pathway”. However, we believe that the information we have included in the “Training methods” section addresses your concerns.

*2. The authors propose that the efficiency of the training methods will be assessed by measuring the number of sessions needed to reach a criterion that is determined by the trainers. However, it is fundamental to know what would be the length (N. of trials? Time?) of a training session. Training sessions of different durations have been shown to produce different outcomes (Demant et al. 2011). The duration or number of trials of the sessions should be somehow standardized.*

Please refer to response to comment #11 of Reviewer #1.

*3. It is likely that both the dogs and the handlers of this study will have extensive experience with mixed methods, but little or no experience with reward methods. I think that this may affect the results. How do the authors plan to take it into account?*

Reward methods have been used consistently in both institutions (PSP and RPara) for more or less 10 years. We do not foresee any issue regarding the experience of handlers with both methods.

*4. Since the evaluation of the dogs’ performance in the test is somewhat subjective, I warmly recommend the observer that will score the dogs’ performance to be blind with regard to the treatment received by the dog, to avoid a biased judgment.*

Please refer to response to comment #19 of Reviewer #1.
