# Supplementary material for: Improving dog training methods: Efficacy and efficiency of reward and mixed training methods
Source: PLoS One. 2021 Feb 19;16(2):e0247321. doi: 10.1371/journal.pone.0247321 (PMC7895348; doi:10.1371/journal.pone.0247321)
Supplement: Attachment — Submitted filename: Response to Reviewers.docx [file pone.0247321.s004.docx]

RESPONSE TO REVIEWERS

Once again, we would like to thank the reviewers for the constructive criticism. The current version of the manuscript addresses the last round of comments by Reviewer #2. We detail them in what follows.

“Since flexibility is allowed for the trainers to choose which actual rewards and punishments to use, I strongly recommend that in the data collection the authors include a list of the rewards and punishments actually used by the trainers, because these may play an important role in determining the outcome of the training.”

We agree with the reviewer. We thought this was made clear in the previous version of the manuscript when we wrote, in lines 265-268, “The video recordings of the training sessions (…) will also allow for the analysis of handler behavior and other aspects of training such as the frequency, type and timing of the stimuli applied”. However, in order to leave no doubt about this idea, we added the current sentence in line 297: “This will be used to generate a list of all the conditioning procedures actually used by each handler during training”.

“Line 62: a parenthesis is missing from the reference.”

Thank you for noticing that! It is now corrected.

“Lines 151-152: This sentence describing the subjects' previous experience may better fit above (e.g. in line 138). Then you can continue with the more detailed description of similar behaviours previously learnt by the dogs.”

We followed the reviewer’s suggestion and moved this sentence to the beginning of the paragraph (Line 139).

“Lines 256-257: What will be the criterions for scoring the performance? E.g., speed of execution? Latency to execute? Detailed criterions may help reducing the subjectivity level of this judgment.”

We understand the reviewer’s concern here. In a first draft of our qualitative scoring system, we actually had more criteria detailed for each exercise. However, after carefully and thoroughly discussing it with the members of the author team who work very closely and in practice with this type of scoring systems (Ângelo Aráujo and André Fonseca), the end result was the system we proposed in the previous version of the manuscript (Annex S1). Given that the international experts on working dog training that will evaluate dog performance for our study are also familiar with (and actually implement in practice) this type of system, we feel this is actually the best approach for our research.
